# A New Model of Salivary Pacemaker—A Proof of Concept and First Clinical Use

**DOI:** 10.3390/medicina59091647

**Published:** 2023-09-12

**Authors:** Cristian Funieru, Dan Ștefan Tudose, Bogdan Dobrică, Mihai Săndulescu, Ion Alexandru Popovici, Emil Ioan Slușanschi, Sorin Mihai Croitoru, Daniela Vrînceanu, Bogdan Bănică, Mihnea Ioan Nicolescu

**Affiliations:** 1 Preventive Dentistry Department, Faculty of Dentistry, Carol Davila University of Medicine and Pharmacy, 4 Eforiei, 050037 Bucharest, Romania; cristian.funieru@umfcd.ro; 2 Computer Science and Engineering Department, Faculty of Automatic Control and Computer Science, Politehnica University of Bucharest, 313 Splaiul Independenţei, 060042 Bucharest, Romania; dan.tudose@upb.ro (D.Ș.T.); emil.slusanschi@upb.ro (E.I.S.); 3 NanoSAE Research Centre, Faculty of Physics, University of Bucharest, 405 Atomiştilor, 077125 Bucharest-Măgurele, Romania; bogdan@3nanosae.org; 4 Department of Implant Prosthetic Therapy, Faculty of Dentistry, Carol Davila University of Medicine and Pharmacy, 17-23 Plevnei, 010221 Bucharest, Romania; mihai.sandulescu@umfcd.ro (M.S.); ion.popovici@umfcd.ro (I.A.P.); 5 Department of Machine Tools and Manufacturing Systems, Faculty of Industrial Engineering and Robotics, Politehnica University of Bucharest, 313 Splaiul Independenţei, 060042 Bucharest, Romania; sorin.croitoru@upb.ro; 6 ENT Department, Faculty of Medicine, Carol Davila University of Medicine and Pharmacy, 169 Splaiul Independenței, 050098 Bucharest, Romania; 7 Oral and Maxillofacial Surgery Department, University Emergency Hospital Bucharest, 169 Splaiul Independenţei, 050098 Bucharest, Romania; bogdan.ddr@gmail.com; 8 Histology Division, Faculty of Dentistry, Carol Davila University of Medicine and Pharmacy, 8 Eroii Sanitari, 050474 Bucharest, Romania; 9 Radiobiology Laboratory, Victor Babeș National Institute of Pathology, 99-101 Splaiul Independenţei, 050096 Bucharest, Romania

**Keywords:** xerostomia, salivary secretion device, dental implant

## Abstract

*Background and Objectives*: Saliva is of utmost importance for maintaining oral health. Management of saliva flow rate deficiency recently includes salivary neuro-electrostimulation. The aim of this paper is to present a new model of salivary pacemaker—the MICROSAL device (MD), an intelligent, miniaturized, and implant-supported oral device used for salivary stimulation. *Materials and Methods*: This report presents the development, calibration, and first clinical tests which involved the MD. The novel features of this device are the pH sensor and the fact that it communicates with the patient’s smartphone, where oral wetness and pH are graphically exposed. Saliva samples were taken before and after the MD was used on a 68-year-old patient suffering from post-irradiation xerostomia, and albumin and total protein were analyzed. *Results*: The device uses up to 3 V and time intervals of 2 s seconds for stimulation. The total volume of all saliva samples collected during the clinical trial was almost seven times higher after the device was used. Albumin decreased from a maximum of 0.15 g/dL to 0.04 g/dL, and total proteins from 0.65 g/dL to 0.21 g/dL, after salivary stimulation. *Conclusions*: The MD increased saliva secretion of the patient, and we are confident it will be a good solution for future management of salivary gland hypofunction.

## 1. Introduction

Saliva is known to play a pivotal role in maintaining oral health due to its antimicrobial activity and action to keep a neutral pH range or provide the necessary amount of calcium and phosphate ions for teeth remineralization [1,2]. About 90% of saliva is produced by the three pairs of major salivary glands, and the rest comes from the 600–1000 minor glands [3,4]. The salivary flow rate varies from 0.3–0.4 mL/min (unstimulated) to 1.5–2.0 mL/min (stimulated) [4]. While salivary gland hypofunction (SGH) is a functional impairment that leads to a low saliva flow rate, i.e., less than 0.1–0.2 mL/min (unstimulated) and less than 0.7 mL/min (stimulated), xerostomia is rather a perception or a complaint of dry mouth, which may or may not have a background of saliva flow rate deficiency [2,5]. The treatment guidelines depend on a correct diagnosis of xerostomia, SGH, or both. The treatment of xerostomia is based mostly on dry mouth symptom relief, such as the usage of sugar-free chewing gum and candies or regular consumption of water. The treatment of SGH involves two strategies: (1) the extrinsic approach, which involves artificial saliva substitutes, but the efficiency of which is limited, and (2) the intrinsic approach, based on the stimulation of saliva secretion, which is preferred [1,4,6]. Therefore, muscarinic agonists like pilocarpine and cevimeline are mostly used, as well as substances which can also stimulate salivation through taste perception such as citric acid or lemon juice. However, muscarinic agonists have several side effects, including sweating, rhinitis, nausea, and asthenia, whereas citric acid solutions may lead to mucosal irritation or enamel mineral losses [4].

The problems with different pharmacologic agents have opened up a new chapter for the intrinsic approach: salivary neuro-electrostimulation. Initially, there were a few attempts at using the transcutaneous electric nerve stimulation (TENS) method. TENS uses external devices with electrodes put on the facial skin around the parotid gland area, which leads to an increased parotid salivary flow rate. TENS devices yielded positive responses in cases with low but non-zero baseline salivary flow rates, but their effect lasted only for 30 min from the moment of applying. However, TENS devices exhibited some side effects, including facial muscle twitching, superficial anesthesia or itching, and a decreased effect of saliva stimulation in elderly people, which leads to low applicability [7,8,9,10,11]. The first attempt with intraoral devices was made in 1986 in the USA: the Salitron device (Biosonics, Fort Washington, PA, USA) was quite big and consisted of a large power source and an oral piece with two electrodes. It was uncomfortable for the patients, and also expensive [12,13]. An easier-to-use device with a newer design is SaliPen (Saliwell Ltd., Harutzim, Israel/Saarbrücken, Germany), with a similar operating system [14]. The GenNarino (Saliwell Ltd., Harutzim, Israel/Saarbrücken, Germany) is a removable intraoral second-generation appliance intended to increase saliva secretion. It has a wetness sensor, electrodes (in contact with oral mucosa from the lower third molar area), a microprocessor, batteries, and a remote-control receiver. The device was controlled by the patient using a remote control. The Saliwell Crown (Saliwell Ltd., Harutzim, Israel/Saarbrücken, Germany) represents the third and the last generation of oral devices, miniaturized and supported by a dental implant placed in the lower third molar area. Its electronic components are similar to those of GenNarino™ (Saliwell Ltd., Harutzim, Israel/Saarbrücken, Germany) [6,12].

Until now, the efforts of medical specialists have focused on limitation of the impact on salivary secretion during radiation therapy by modulating the dose administered around salivary tissue. This strategy has in some cases resulted in lowering the efficacy of radiotherapy and increasing the risk of cancer recurrence [15]. There are also other common causes for xerostomia, such as various medications, immune mediated-disorders (Sjogren syndrome), diabetes mellitus, depression, and Alzheimer’s disease [1].

This paper presents a new model of salivary pacemaker—the MICROSAL device (MD), which is an implant-supported third-generation device with a different design. The MD is an intelligent device, which includes two new models of nano-sensor (wetness and pH) and which is capable of stimulating the salivary flow rate depending on mucosal wetness and oral pH value. The salivary parameters are sent in real time to the patient’s smartphone using the Bluetooth Low Energy technology (BLE) and a specially designed app. The main innovations of the MICROSAL device compared with other devices mentioned in the literature, especially third-generation devices, are:

− Management of saliva flow rate depending on the pH value.
− Two brand-new nano-sensor prototypes (wetness and pH).
− BLE technology makes it possible to collect a lot of data from the remaining saliva.
− Use of an app for monitoring and management of saliva flow rate by both the dentist and the patient.

## 2. Materials and Methods

**Figure 1:**
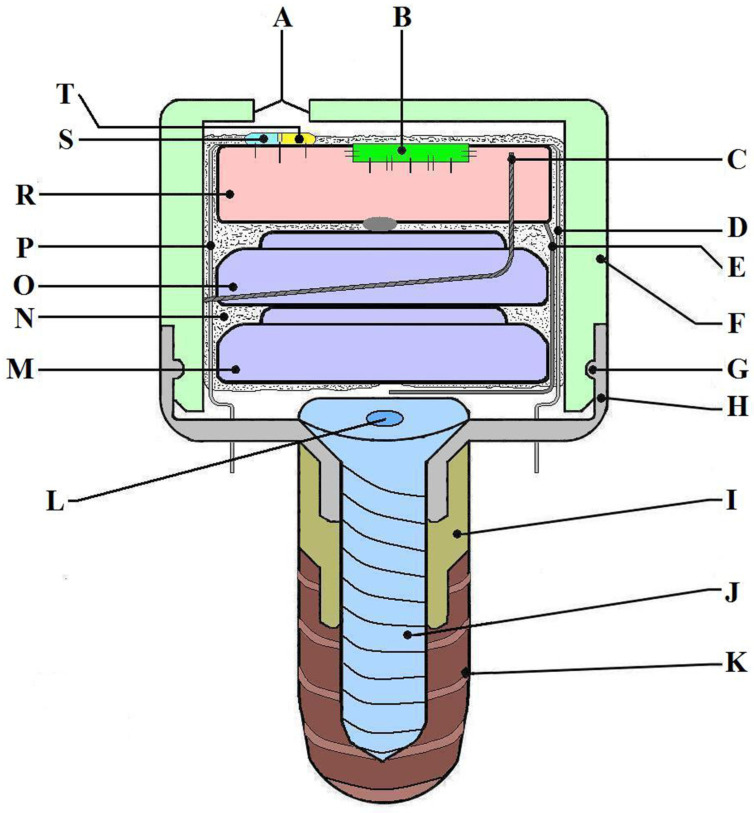
The MICROSAL device (MD) scheme: (A) main hole from the upper case (made for the sensors to be in contact with saliva), (B) microcontroller, (C) mini-antenna used for sending signals to the smartphone via Bluetooth technology, (D) one of the electrodes used for stimulation, (E) a wire which makes the connection to the batteries and activates the system when it is put in its case, (F) the upper part of the case, made from polypropylene, (G) the fastening elastic system (clips), (H) the downer part of the case, made from titanium alloy, (I) a metallic extension, also made from titanium alloy, (J) implant central cover screw, (K) the dental implant, Tehnomed system™, (L) the central part of the screw, (M, O) batteries, (N) the coating material (PanacolVitralit 2004 F), (P) the second electrode for stimulation, (R) printed circuit board (PCB), and (S, T) wetness and pH sensors.

**Figure 2:**
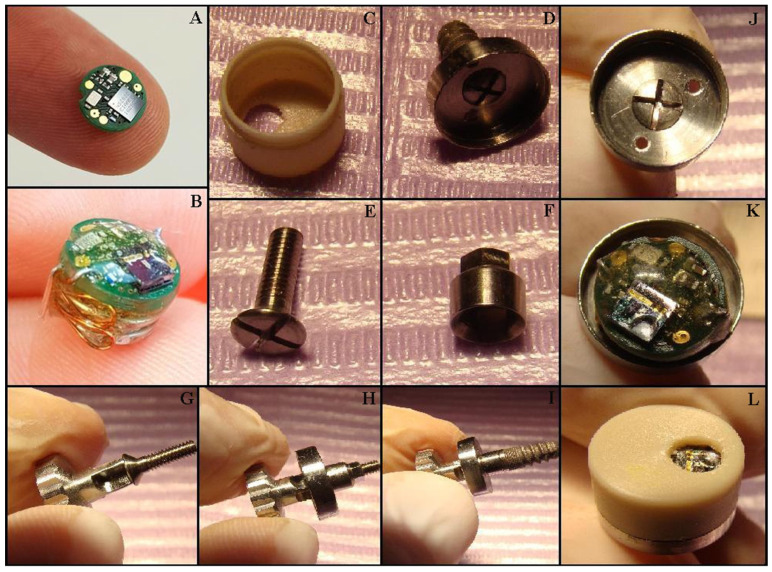
Mounting the MICROSAL device (MD): (**A**) the printed circuit board (PCB); (**B**) the MD out of its case; (**C**) the upper part of the case, made from polypropylene; (**D**) the lower part of the case, made from titanium alloy; (**E**) the implant central cover screw; (**F**) the metallic extension, made also from titanium alloy; (**G**) the central screw and the screwdriver, Tehnomed™ system; (**H,I**) the titanium case and the metallic extension are fixed on a dummy dental implant; (**J**) the lower part of the case and the two orifices for the electrodes; (**K**) the inside of the MD; (**L**) the operation is complete after the upper case is fixed.

Device description: The MD was developed by an interdisciplinary research team including dentists, oral and maxillofacial surgeons, ENT specialists, engineers, programmers, and physicists. The MD’s main role is to stimulate the salivary glands using electrical impulses to increase salivary flow rate. It is designed to have minimal-to-no impact on daily life and patient activities and has a very small size, comparable to that of a molar. The MD is fixed on a dental implant (Tehnomed™ system, Tehnomed, Bucharest, Romania) (Figure 1), and its main components are a printed circuit board (PCB) (Figure 1R and Figure 2A), a microcontroller (Figure 1B), two silver-oxide batteries (Figure 1M,O), two nano-sensors for wetness and pH (Figure 1S,T), and two microelectrodes for stimulation (Figure 1D,P). All components are encapsulated in a coating material, namely Panacol Vitralit 2004 F (PV), based on epoxy resins (Panacol-Elosol GmbH, Frankfurt, Germany) (Figure 1N and Figure 2B), and afterward the system is placed in a hermetic case.

The case consists of two parts: an upper threaded lid, made of polypropylene (Figure 1A and Figure 2C), with rounded edges and a small hole for the sensors to be in contact with the saliva and the lower housing, made of titanium alloy (Figure 1H and Figure 2D,J), with two small orifices for the electrodes to be in close contact with the oral mucosa around the implant. The two parts are fixed together by an elastic system (clips). Note that all the external parts are made from biocompatible materials (polypropylene, titanium alloy, or epoxies). The device uses a wireless communication protocol to send data and receive commands from the patient’s smartphone, thus minimizing the inconvenience caused to the patient when monitoring, tracking progress across the day, and transmitting alerts. The microcontroller has very low power consumption, which provides connectivity to the sensors and a way to communicate to the exterior. NRF52832 is a system-on-a-chip that offers these facilities (Nordic Semiconductor, Trondheim, Norway) and a very small footprint, which makes itself ideal for low-power and small-size applications. The package of the microcontroller comes within a 3 × 3 mm footprint, and the CPU has a minimum current consumption of 300 nA in deep sleep mode. The fact that we can periodically “wake” the device from a low-power state, measure sensor data, and then log them internally and/or send them via the Bluetooth link to a mobile app leads to a very long operational lifetime for the device. The device uses two SR65 silver oxide 14.5 mAh batteries placed in the largest section of the system. Due to biocompatibility, size, and accuracy constraints, the chosen pH sensor is an N-type ion-sensitive field-effect transistor (ISFET) with a 6 μm wide silicon oxide source channel separated by a thin 65 nm silicon nitride layer from the oral cavity, with an integrated silver–silver chloride reference electrode conformally coated with PV. The humidity sensor is a resistive sensor with two magnetron-sputtered 300 nm thick silver electrodes laid through on top of a 700 nm silicon oxide layer sharing the same N-type substrate as the pH sensor. The MICROSAL app is designed for version 7 of the Android mobile operating system (Open Handset Alliance, Mountain View, CA, USA). The software controls the hardware components so as to generate an electrical stimulation waveform and responds to Bluetooth commands that dictate the waveform parameters.

The app allows two kinds of users: the “passive user” (the patient), who can graphically follow in real time the humidity level and the pH values and see any battery alerts or other errors that might lead to a dental appointment, and the “active user” (the dentist), who can allow a professional to configure the waveform parameters, visualize the signals coming from the pH and humidity sensors, and validate the times and dates of dental appointments. The MD can be programmed for automatic electrical stimulation in accordance with data from the two sensors.

The MD mounting procedure is straightforward: firstly, the healing abutment is removed from the dental implant using a Tehnomed (Tehnomed, Bucharest, Romania) screwdriver. Afterward, a screw (Figure 1J and Figure 2E,G) fixes the lower part of the case (Figure 1H and Figure 2H) first onto a metallic extension also made from titanium alloy (Figure 1I and Figure 2F) and then onto the dental implant (Figure 1K and Figure 2I). Note that the metallic extension is custom-made according to the oral mucosa thickness around the dental implant. The micro-system is then placed in the lower case (Figure 2K), carefully passing the electrodes (coated in PV except the tips) through the two orifices. Finally, the upper case is fixed with the clips inside (Figure 2L). The connection between the two parts of the case is very strong, and they can be separated only by the dentist using a metallic instrument.

### Procedures for In Vitro and Clinical Tests

In-vitro testing for the two nano-sensor prototypes was performed in a specially designed enclosure, filled with commercially available saliva, with temperature and humidity closed-loop-maintained using a micro-controller actuating an ultrasonic atomizer, a Peltier condenser, a resistive heating element, and a fan to closely simulate oral cavity conditions. The chemical stability of the sensors was confirmed by the very low variation (under 0.4%) in peak intensity in liquid chromatography–mass spectrometry analysis of the immersion saliva with and without the sensor. The clinical protocol was approved by the Ethics Committee for Scientific Research of the “Carol Davila” University of Medicine and Pharmacy, Bucharest, Romania (Approval code #39/29.09.2014), and all patients signed an informed consent form. In order to obtain a complete and functional electronic system, a macro version of the device was developed and tested by our engineers. The null hypothesis was that salivary volume would remain the same after the device had been mounted into the oral cavity. This device was used in a clinical test to adjust it and certify that the MD’s operating principles will lead to an increased saliva flow rate. Thus, two round dental probes were connected to the macro system’s electrodes and were put in contact with the lingual and vestibular mucosa in the third molar area of a volunteer patient (45-year-old, woman). This test was necessary to determine the perfect balance between electrical impulses (pulse length, frequency, and voltage), saliva flow rate (measured using graduated containers), and the patient’s comfort (the patient must not feel any pain). The macro device was miniaturized and further optimized by our technical team. The final version received the name MD and has undergone preliminary testing in a clinical case.

Clinical tests: The MD was clinically used and tested on a patient (a 68-year-old man) suffering from post-irradiation xerostomia. Six samples of saliva were collected (out of which the last three were collected with the MD fixed on the dental implant) in the morning between 9 and 10 a.m., before eating. The patient was asked to spit for 5 min for each probe. The saliva flow rate was measured using plastic graduated containers forming Saliva Check Buffers (GC Corporation, Tokyo, Japan). Afterwards, the saliva samples were sent to the biochemistry lab for albumin and total protein dosages. Saliva was transferred in sterile tubes and centrifuged for 10 min at 3000 rpm to remove bacterial and cellular debris, and the supernatant obtained was used for the next steps. Both protocols, for albumin and total proteins, used analyzing kits (Dialab GmbH, Vienna, Austria) and a biochemistry analyzer, Zevitt 120 (Salucea, Hoeven, The Netherlands), with a working protocol of 500 µL reagent + 5 µL supernatant and incubation for 5 min at 37 °C. The principles were based on the colorimetric method: the proteins formed a blue-purple stain with copper ions in an alkaline solution; the absorbance of the colored complex formed was directly proportional to the protein concentration of the sample with a reading absorbance of 540 nm. On the other hand, the albumin assay was based on the interaction between Bromocresol Green and albumin, forming a chromophore that can be detected at 620 nm. The color intensity was directly proportional to the albumin concentration in the sample.

## 3. Results

### 3.1. In Vitro and Clinical Tests

#### 3.1.1. Sensor Testing

**Figure 3:**
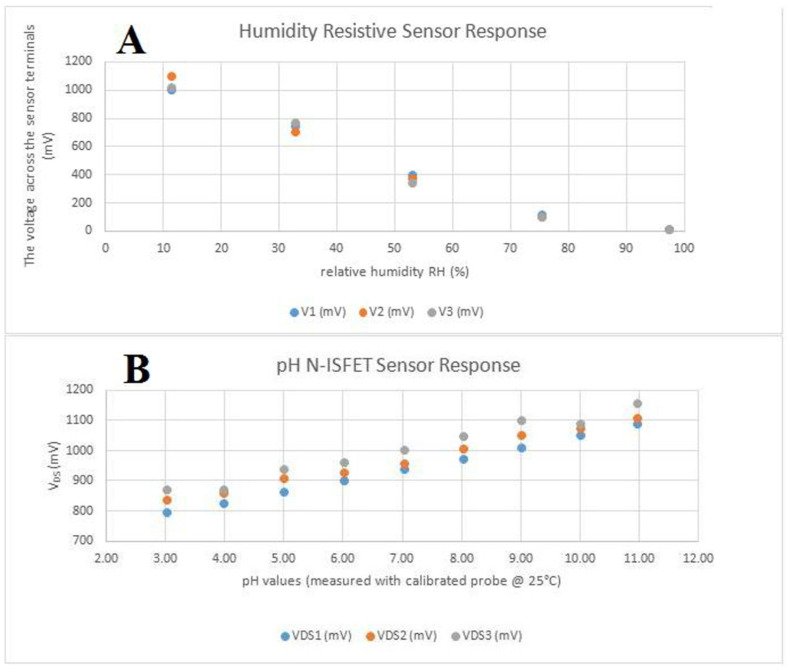
In vitro tests of the two sensors: (**A**) the humidity-resistive sensor response; (**B**) the pH N-ISFET sensor response.

Sensors were tested separately from the main device. The humidity sensor has a quasi-linear, inversely proportional response to the relative humidity of the environment as observed in Figure 3A. When connected as the bottom part of a resistive divisor, together with a 1 MΩ resistor with a 3.3 V power supply, the variation is in the range of 0 to 1.3 V, which makes it appropriate to be measured by the analog-to-digital converter from the micro-controller, using the internally available reference only. pH sensors were properly calibrated using an Atlas Scientific lab-grade pH probe and appropriate calibration solutions. The sensor responded quasi-linearly, directly proportional to the change in the pH level, as observed in Figure 3B, again in the analog-to-digital-converter-suitable (0–1.3 V) interval. For stability testing, a batch of pH sensors was immersed in a standard solution with pH 7(±0.2) and we observed a consistent drift, caused by the reference electrode’s interaction with the environment and Si_3_N_4_ layer saturation. The drift was software-compensated.

#### 3.1.2. Clinical Trial

In the clinical trial performed for testing the macro device, we used, for stimulation, previously published electrical parameters: a 4–10 ms pulse length, 0.1–8 Hz frequency, and three variants of voltage (3 V, 3.35 V, and 5 V) [16]. We then analyzed the patient’s saliva flow rate and painful sensitivity and, considering the technical possibilities for the miniaturized device, we changed some parameters and chose the following sequence for electrical stimulation: 1 ms pulse length, 10 Hz frequency, and a voltage up to 3 V. The sequences of stimulation measured two seconds each followed by two-second pauses. These are the parameters used by the MD for increasing the saliva flow rate.

A 68-year-old male was selected for the first clinical report with the MD. He was diagnosed in 2002 with low-grade (G3) nasopharyngeal squamous cell carcinoma, T_4_N_0_M_x_, according to the classification of the Union for International Cancer Control [17]. He followed a combined therapy of megavoltage conventional radiation (total dose 70 GY) targeted on the tumor and lymph nodes and three doses of 100 mg/m^2^ of cisplatin. The patient had a good recovery, and the treatment was quite efficient, but lack of saliva remained a real problem for his quality of life. The total volume of the three saliva samples before stimulation was approximately 0.5 mL, which corresponds to a 0.03 mL/min saliva flow rate. Due to the very small amounts of saliva in these probes, the true volume was determined in the biochemistry lab. The MD could be easily mounted because the patient was completely edentulous. The patient had not performed chewing while wearing the device, and mandibular movements appeared normal under examination.

**Figure 4:**
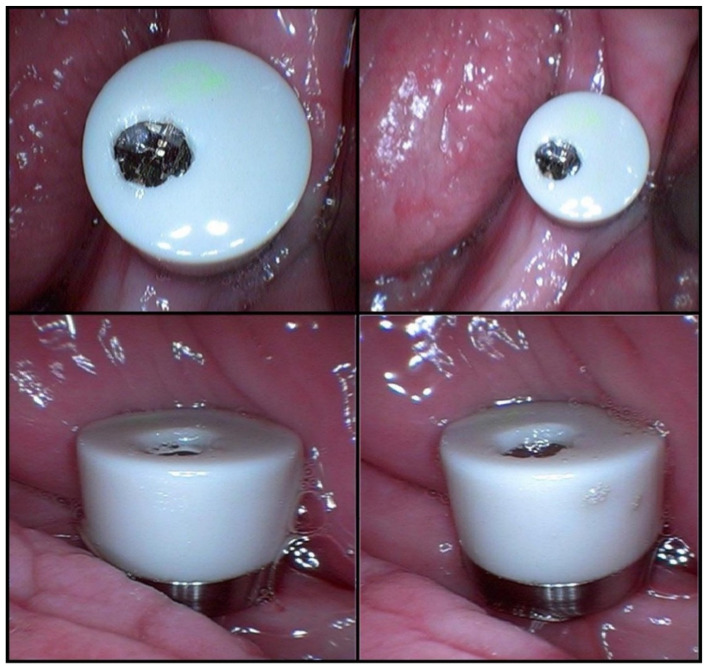
The MICROSAL device (MD) in the oral cavity.

The three samples taken after salivary stimulation had 1 mL, 1 mL, and 1.5 mL of saliva. The low concentrations of albumin and total proteins in the last three saliva samples may have been caused by dilution (a larger volume of saliva) (Table 1). The MD was mounted in the oral cavity after the first three samples (Figure 4).

## 4. Discussion

The MICROSAL device (MD) is a result of interdisciplinary research, which involved a lot of expertise in both medical and technical areas. All parts of the device except the batteries, namely the PCB, sensors, and housing had their own projects and were developed from the beginning. The clinical test results are an argument for electro-stimulation therapy of the salivary glands being a solution for patients suffering from SGH. However, any therapy based on salivary gland stimulation depends on how functional the glandular tissues are. We know that drugs like pilocarpine require functional residual salivary gland tissues making possible an increase in the saliva flow rate [4]. The electrostimulation method’s efficiency also depends on healthy residual salivary gland tissues, but it seems that this method is also associated with the stimulation of the autonomic nervous system, which leads to trophic effects and regeneration of functional salivary parenchyma [12].

One of the strong points of the MD is the continuous stimulation of saliva secretion, which is a real advantage over the TENS or the first generation of oral devices. The MD obviously has a small volume compared with the first and the second generations of intraoral devices. It is also an implant-supported device like the Saliwell Crown™ (Saliwell Ltd., Harutzim, Israel/Saarbrücken, Germany), placed in the lower third molar area, which requires a special clinical precondition, i.e., the absence of one the lower third molars, if not the second molar as well. Moreover, the absence of the third upper molar from the same side may prove useful, since the MD lacks any occlusal function. The MD is recommended for patients who lack both third molars on one side (right or left), which is quite common in elderly patients. If the patients have teeth, the occlusal relationships can be adapted by the dentist. If the patients have removable dentures, it is recommended to make new ones with the implant analog included in the dental cast and an empty case device mounted on it. It is possible that MD touches the superior alveolar mucosa if the patients have no teeth or removable dentures. It is recommended for edentulous patients to wear dentures made after the device was mounted.

The MD also brings some new features and technologies. The first novelty is the pH sensor, which was designed to detect acidity values in the oral cavity. When the pH decreases, more saliva is needed for buffering and for maintaining a balanced oral environment. The device is intended to increase the saliva flow rate in case of lack of saliva in accordance with data received from the wetness sensor. In addition, saliva secretion is further increased when the patient has both a small amount of saliva and a low pH value. Note that both sensors have nanometric dimensions. The previous devices, namely the GenNarino™ (Saliwell Ltd., Harutzim, Israel/Saarbrücken, Germany) and Saliwell Crown™ (Saliwell Ltd., Harutzim, Israel/Saarbrücken, Germany), also use wetness sensors [6,12,18]. Miniaturized or nano-sensors have previously been used for saliva monitoring and proved useful in detecting salivary glucose or oral cancer biomarkers [19,20]. Another new element is the option of programming the device for self-regulation of electrical stimulation according to wetness and pH values using an algorithm, which makes it “responsible” for an artificial saliva secretion reflex. A real advantage of the MD is continuous secure communication with the patient’s smartphone via an app specially designed for Android. Thus, the patient can follow in real time the wetness and pH values, which are graphically exposed. The patient and the dentist may also see alerts on their smartphones caused by different malfunctions. For example, a battery alert leads to a dental appointment requested by the patient and validated by the dentist. The dentist can also be given permission to remotely monitor stimulation and manage some of the problems by checking or adjusting its parameters. This component of patient–doctor communication was added for increasing treatment quality and for quick troubleshooting. In any case, medical apps are becoming more and more widely incorporated and used in medicine, especially in clinical practice [21]. The development of this device could benefit, in the future, patients with autoimmune diseases like Sjogren Syndrome, a pathology involving an immune imbalance affecting the salivary glands and requiring continuous systemic observation [22]. If no third molars are present on any side, the dentist can choose to mount the MD on the side with the salivary gland most able to produce saliva (certified by the CT and preliminary clinical tests). However, when only one salivary gland (submandibular) has problems with saliva secretion or, in particular, if one of the two glands has been surgically removed, the remaining gland reacts and produces a compensating volume of saliva close to normal values. The regenerative properties of salivary glands [23,24] and their putative underlying mechanisms [25,26,27] are already under thorough study. It appears that the new SARS-CoV-2 virus is also affecting the salivary glands, and a statistically significant increase of about 30% in the reporting of xerostomia during hospitalization was observed [28]. Another category of patients that could benefit from the development of this device are those with poor oral hygiene due to neurological conditions like Parkinson’s disease [29]. Clearly, the greatest impact for our device will be among patients undergoing radiation therapy for head and neck carcinomas, by reducing xerostomia and improving their quality of life [30]. The future of biotelemetry devices will yield an acceleration of diagnosis and disease monitoring, mostly benefiting patients but also medical personnel [31].

## 5. Conclusions

The MD is at the stage of first clinical results and of course a larger clinical investigation will be needed to validate its usage on a large scale. Further studies will be needed to analyze the long-term use of the implant and possible problems due to electrical impulses or any other long-term issues. What we do know is that the device increases saliva flow rate immediately after it is mounted in the oral cavity, that the patient feels no pain, and that its very strong attachment on the dental implant will decrease the chance of any accident or local trauma. The MD seemed to increase saliva flow rate in the case of our patient with SGH due to post-irradiation therapy. Based on this first clinical result, we look forward to the future, where the MD will be a good solution for more patients suffering from SGH. The MICROSAL app is currently only available for Android, (Open Handset Alliance, Mountain View, CA, USA) but an iOS version will be developed in the future.

## 6. Patents

The work reported in this manuscript is based on the Patent Application no. (11) 133,137 A2 2019, RO-BOPI 3:22.

## Figures and Tables

**Table 1:** Albumin and total protein dosages before and after saliva stimulation with the MICROSAL device (MD).

Sample	Albumin (g/dL)	Total Protein (g/dL)
No. 1	0.15	0.643
No. 2	0.18	0.652
No. 3	0.14	0.459
No. 4 *	0.04	0.250
No. 5 *	0.04	0.278
No. 6 *	0.03	0.217

* Saliva samples after stimulation with MD.

## Data Availability

The data presented in this study are available on request from the corresponding author. The data are not publicly available due to undergoing patenting.
